# Supplementary material for: Mechanistic insights into RNA surveillance by the canonical poly(A) polymerase Pla1 of the MTREC complex
Source: Nat Commun. 2023 Feb 11;14:772. doi: 10.1038/s41467-023-36402-6 (PMC9922296; doi:10.1038/s41467-023-36402-6)
Supplement: Supplementary file 1 — Supplementary Information [file 41467_2023_36402_MOESM1_ESM.pdf]

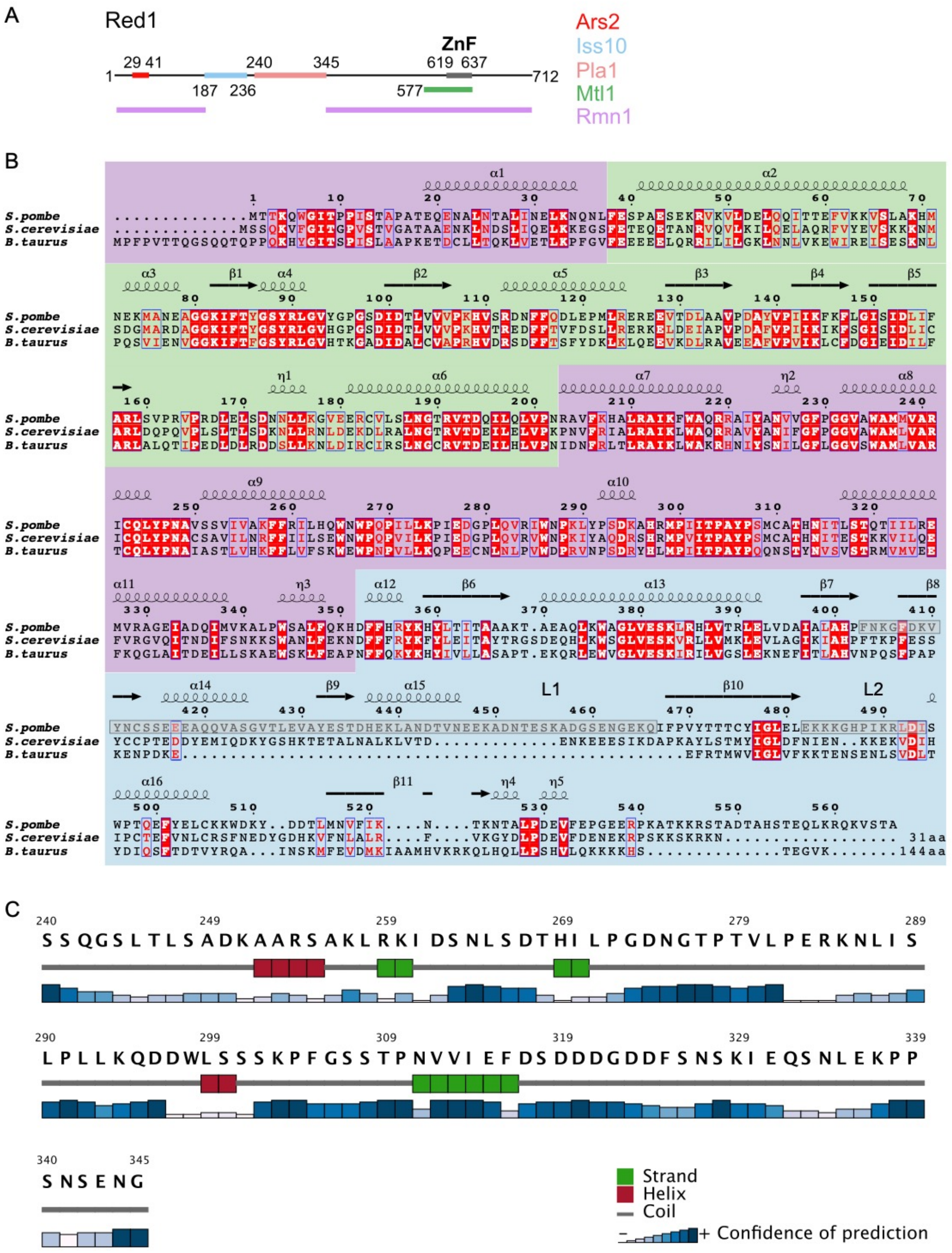

Supplementary Figure 1. Interaction regions of MTREC components within Red1, sequence alignment of Pla1 and secondary structure prediction of Red1$_{240-345}$

(A) Schematic representation of different regions of Red1 interacting with other components of MTREC. (B) Sequence alignment of Pla1 from S. pombe and its homologues from *S. cerevisiae* (Pap1) and *B. taurus* (PAP) are shown. Residues which are identical are highlighted in red while those that are highly similar are colored in red and boxed in blue. 31 and 144 amino acids (aa) at the C-termini of Pap1 and PAP are not shown in the alignment. Secondary structure and sequence numbering with respect to Pla1 is shown on top of the sequence alignment. The NTD, MD and RRM domain of Pla1 are highlighted in green, purple and blue respectively. Loops L1 and L2 are marked in grey. (C) Secondary structure prediction of Red1 residues 240-345 using PSIPRED v4.0.[1]

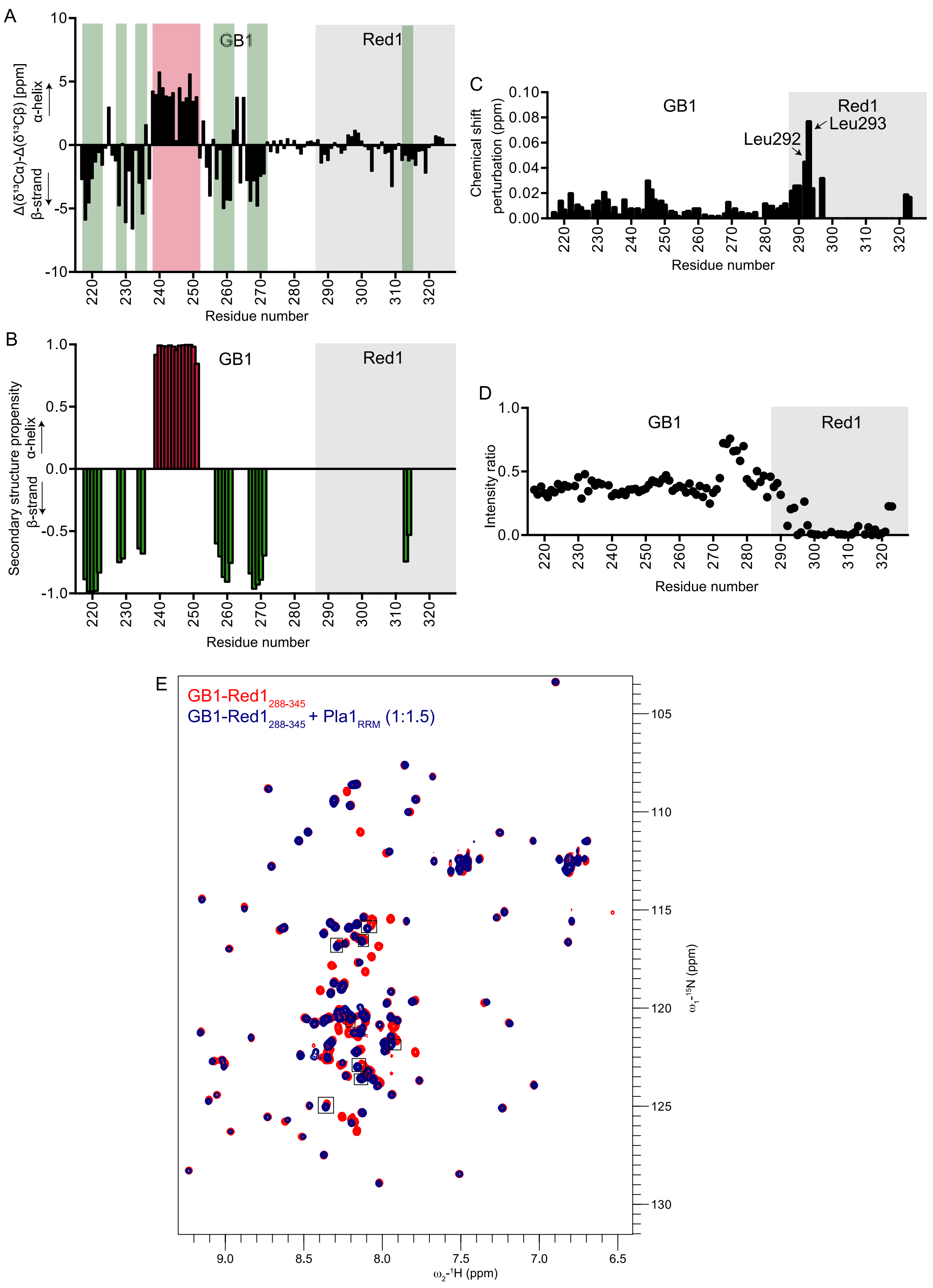

Supplementary Figure 2. NMR analyses of Red1

(A) ^{13}C chemical-shift deviations of C_{α} and C_{β} atoms with respect to random-coil chemical shift values for GB1-taggged $Red1_{288\text{-}322}$ are shown, where positive and negative deviations indicate α-helical or β-stand propensity, respectively. (B) Secondary structure propensity of GB1-tagged $Red1_{288\text{-}322}$ as calculated by TALOS+[2] with α-helices and β-stands colored in red and green, respectively. Secondary structure elements predicted by TALOS+ are also marked in panel A. (C) Chemical shift perturbations arising in GB1-tagged $Red1_{288\text{-}322}$ in the presence of 1.5-fold molar excess of $Pla1_{RRM}$ domain. Residues belonging to Red1 are highlighted in grey. Minor CSPs are observed in the GB1 tag while more pronounced CSPs occur at the N-terminus of Red1 (residues Leu292, Leu293). (D) Intensity ratios of $Red1_{288\text{-}322}$ + $Pla1_{RRM}$ compared to free $Red1_{288\text{-}322}$ are plotted against residue number. (E) Overlay of ^{1}H, ^{15}N-HSQC NMR spectra of GB1-tagged $Red1_{288\text{-}345}$ in the absence (red) and presence (blue) of 1.5-fold molar excess of $Pla1_{RRM}$ domain is shown. Additional Red1 residues which show minor chemical shift perturbations in comparison to GB1-tagged $Red1_{288\text{-}322}$ (**Figure 1D**) are boxed.

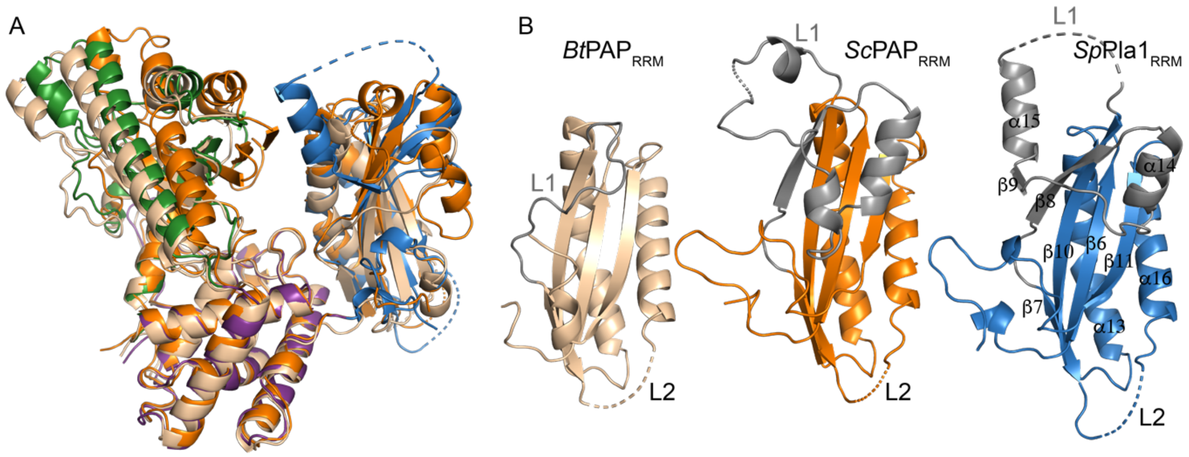

Supplementary Figure 3. Comparison of Pla1 with its homologues

(A) Superposition of crystal structures of Pla1 apo (color scheme based on Figure 1A) with *S. cerevisiae* Pap1 (PDB ID: 2Q66, orange) and mammalian PAP (PDB ID: 1Q79, wheat) based on an alignment on the MD. A displacement of the NTD and RRM domains shows the flexibility of the domain arrangement. (B) Similar views of the RRM domains of Pla1 and its homologues. Positions of loops L1 and L2 are marked, and L1 shown in grey. L1 is severely extended in yeast when compared to mammals, and partially blocks the canonical RNA binding β-sheet interface.

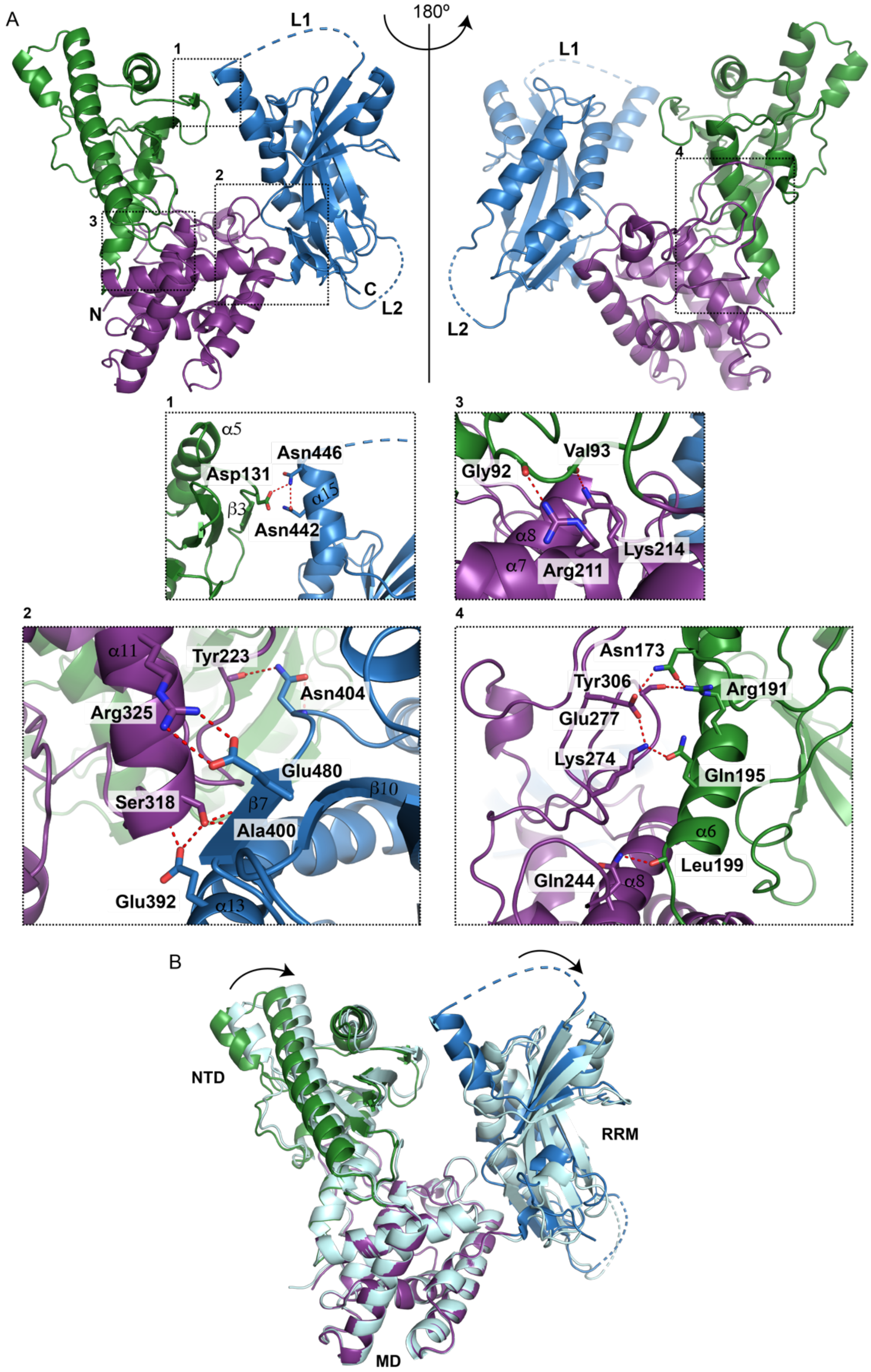

Supplementary Figure 4. Crystal structure of Pla1

(A). Pla1 NTD, MD and RRM domains are held together by four different interfaces which are shown in the insets. Briefly, inset 1 shows that the NTD and RRM domain

are connected via salt bridges between side chains of Asp131, Asn442 and Asn446. Inset 2 shows that the MD and RRM domain are held together strongly by hydrogen bonds between side chain of Asn404 and backbone carbonyl of Tyr223, salt bridges between side chains of Glu480 and Arg325. In addition, a network of interactions between the side chain of Glu392 and backbone amide, side chain of Ser318 and Ala400 backbone is also formed. Interactions between the catalytic domain and the middle domain occur at two interfaces, as shown in insets 3 and 4. The first interface is formed by hydrogen bonds between the sidechain of Arg211 and backbone carbonyl of Gly92, side chain of Lys214 and backbone carbonyl of Val93. The second interface involves a number of interactions mediated via the helix α6 of the NTD. Hydrogen bonds are formed between the side chains of Lys274 and Gln195, side chain of Gln244 and backbone carbonyl of Leu199, side chains of Glu277 and Asn173, backbone carbonyl of Tyr306 and side chain of Arg191. Hydrogen bonds are represented by red dotted lines. (B) Superposition of $Pla1_{FL}$ and $Pla1_{\Delta 14}$ crystal structures based on alignment of the MD. $Pla1_{FL}$ NTD, MD and RRM domain are shown in green, purple and blue, respectively while $Pla1_{\Delta 14}$ is shown in cyan. Displacement of the NTD and RRM domains is shown with arrows.

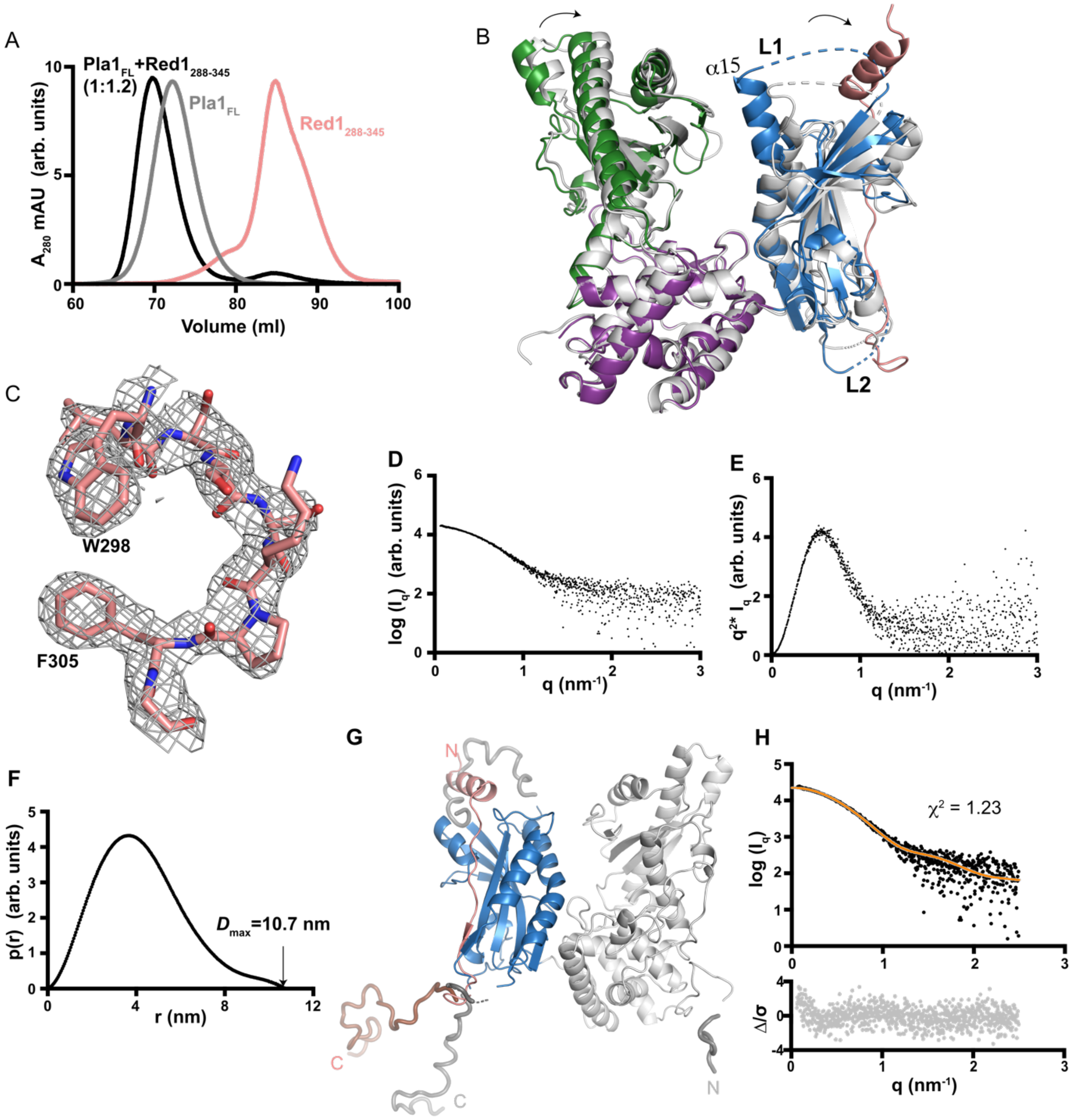

Supplementary Figure 5. Assembly Pla1-Red1 complex

(A) Size-exclusion chromatography profiles of Red1$_{288\text{-}345}$ (salmon), Pla1$_{FL}$ (grey) and Pla1$_{FL}$+Red1$_{288\text{-}345}$ (molar ratio 1:1.2 of Pla1$_{FL}$: Red1$_{288\text{-}345}$, shown in black). A marked change in the retention volume of the Pla1$_{FL}$+Red1$_{288\text{-}345}$ complex compared to the free Pla1$_{FL}$ and Red1$_{288\text{-}345}$ is observed. arb. units represent arbitrary units. (B) Structure superposition of Pla1$_{FL}$ and Pla1$_{FL}$+Red1$_{288\text{-}345}$ complex based on alignment of the MD shows a clockwise displacement of NTD and RRM domains as marked by arrows. (C) 2Fo-Fc map of Pla1-Red1 complex with Red1 residues

Trp298-Phe305 contoured at 1σ shown. (D) SAXS profile plotted as log(I_q) versus q (E) kratky plot and (F) pairwise distribution p(r) curve of Pla1$_{FL}$+Red1$_{288-345}$ complex are presented. arb. units represent arbitrary units. (G) Structure model of Pla1$_{FL}$+Red1$_{288-345}$ complex generated using CORAL[3] to model residues (represented as tubes) disordered in the crystal structure. The NTD and MD are shown in grey while the RRM domain and Red1 are shown in blue and salmon, respectively. (H) Fitting between experimental SAXS and back-calculated SAXS profiles of the Pla1$_{FL}$+Red1$_{288-345}$ complex model generated from CORAL as calculated using CRYSOL[4] and the χ^2 fitting-value of 1.23 are reported. The error weighted residual difference plot is shown at the bottom.

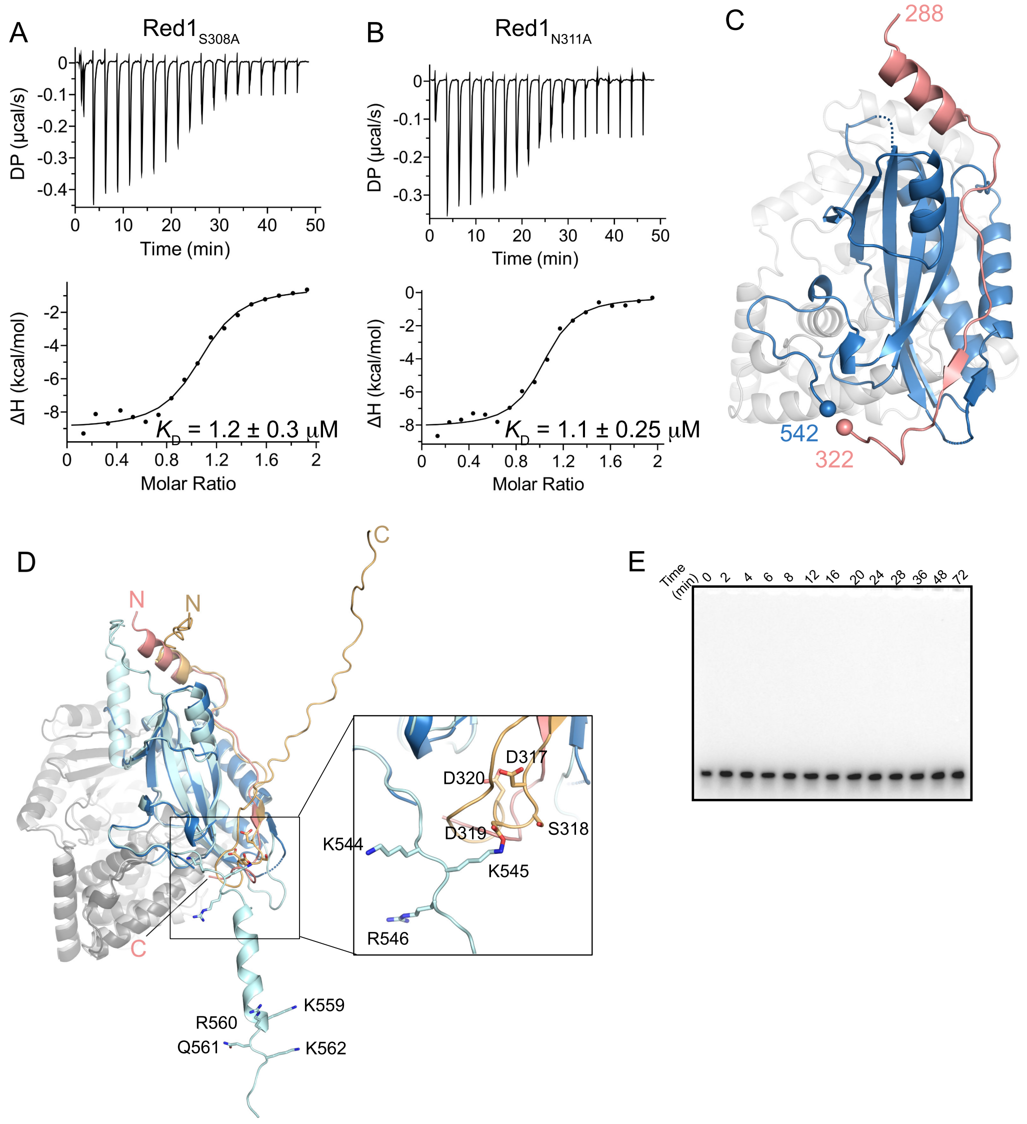

Supplementary Figure 6. Mutational analysis of Pla1-Red1 complex

(A) and (B) Isothermal titration calorimetry experiments with specific point mutants S308A and N311A of Red1$_{288\text{-}345}$ serially titrated into Pla1$_{RRM}$ domain, respectively. The calculated dissociation constant (K_D) from an average of two independent measurements is shown. (C) Spatial proximity between the C$_\alpha$ atoms of Pla1 residue Ala542 and Red1 residue Gly322 which are the last residues at the C-termini of the proteins visible in the crystal structure of the complex are shown as spheres and the distance between them is 3.5 Å. (D) Structure superposition of Pla1-Red1 complex (this study, Pla1$_{NTD}$ and Pla1$_{MD}$ in light gray, Pla1$_{RRM}$ in blue and Red1 in salmon) and the top-ranking model (Pla1$_{NTD}$ and Pla1$_{MD}$ in dark gray, Pla1$_{RRM}$ in cyan and

Red1 in wheat) predicted using ColabFold[5] is shown. Zoomed-in view shows that Pla1 residues K544/K545/R546 are spatially close to Red1 residues D317/S318/D319/D320, in line with our in vitro experiments (Figure 3F). (E) Polyadenylation of a 5'-Cy3 labeled A_{15} RNA primer by a catalytic mutant of Pla1 (D153A) analyzed by 14% denaturing urea PAGE at different time points. A representative gel from two independent experiments is shown.

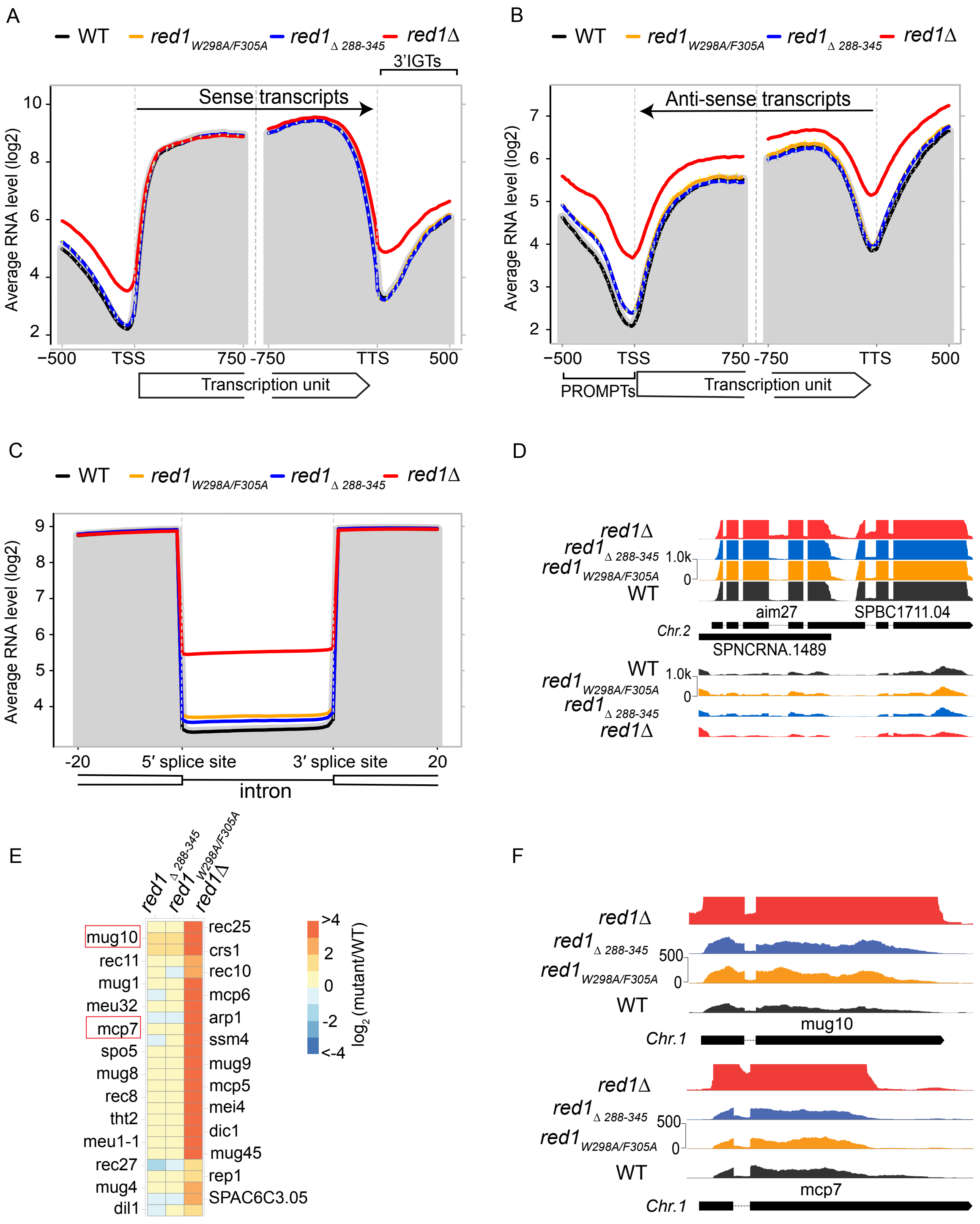

Supplementary Figure 7. Pla1 in the context of the MTREC complex is responsible for degradation of PROMPTs.

(A and B) Metagene profile of sense (A) and antisense (B) RNA levels in the indicated strains for all of *S. pombe* genes. The plots represent the geometric average RNA levels from 500bp upstream to 750bp downstream of the transcription start site (TSS)

and 750bp upstream to 500bp downstream of the transcription termination site (TTS). Solid lines represent the average of two replicates for all indicated strains, with the exception of *red1*Δ which represent a single dataset. Dotted lines represent the individual biological replicates. The grey shading represents the average RNA levels in the WT strain. (C) Metagene profile of sense RNA levels in the indicated strains for all of *S. pombe* introns. The plots represent the geometric average of RNA values from 20bp upstream to 20bp downstream of annotated introns, with the intronic regions scaled to the same lengths for all introns (stretched or condensed). The grey shading represents the average RNA levels in the WT strain. (D) Strand-specific RNA-seq read coverage of a representative set of genes containing multiple introns, in WT and mutant strains. (E) Heat map representation of changes in RNA levels for the meiotic gene cluster in indicated mutant strains compared to WT (log_2 scale). (F) Strand-specific RNA-seq read coverage of a representative set of meiotic genes (Mug10, Mcp7) in WT and mutant strains.

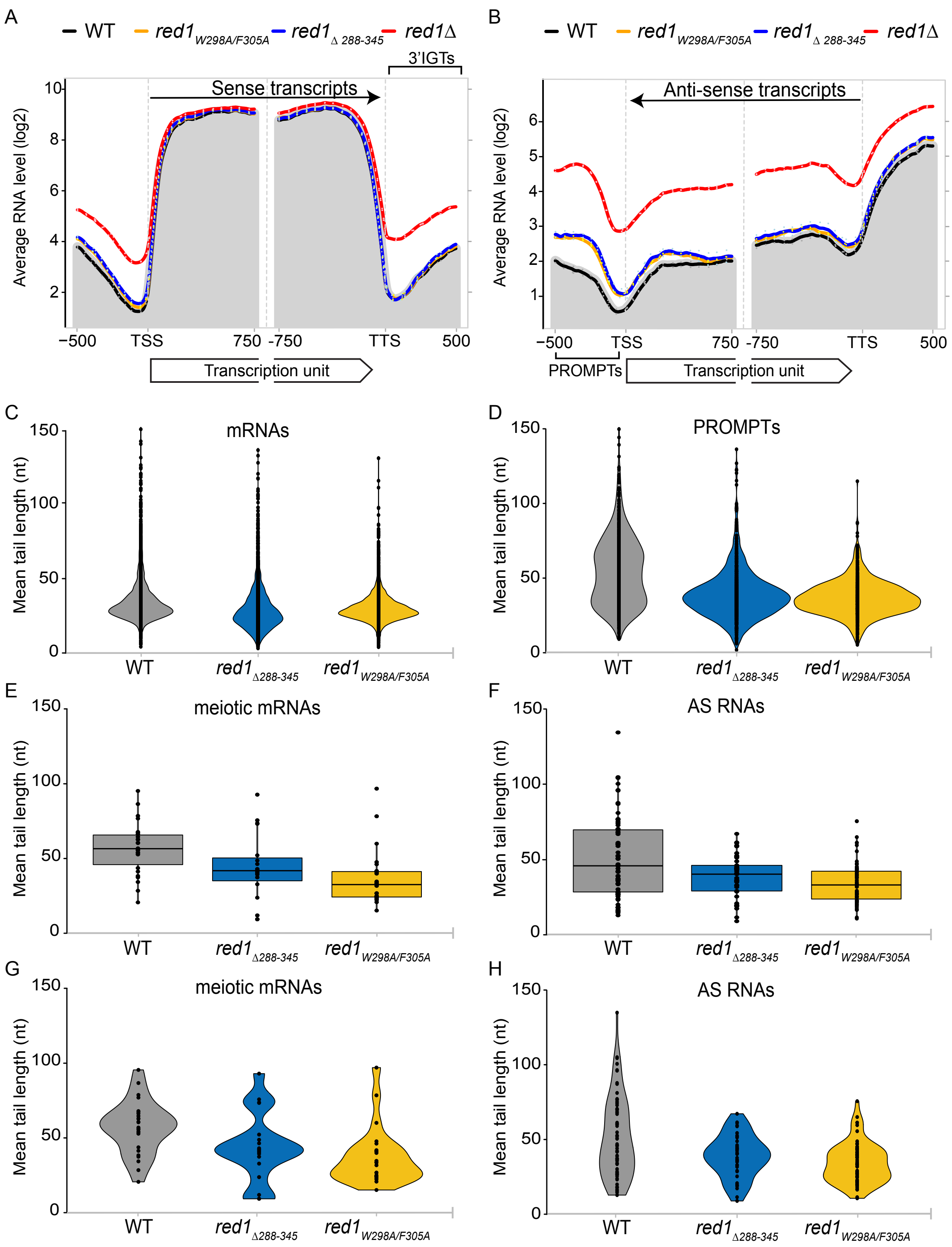

Supplementary Figure 8. Pla1 in the context of the MTREC complex is responsible for extending the poly(A) tail of CUTs.

(A and B) Metagene profile of sense (A) and antisense (B) RNA levels in the indicated strains for a subset of *S. pombe* genes with detectable levels of PROMPTs (2400 genes). The strand-specific total RNA seq experiments were carried out without

poly(A) selection, using random hexamers for reverse transcription (see details in Methods section). The plots represent the geometric average RNA levels from 500bp upstream to 750bp downstream of the transcription start site (TSS) and 750bp upstream to 500bp downstream of the transcription termination site (TTS). Solid lines represent the average of two replicates for all indicated strains. Dotted lines represent the individual biological replicates. The grey shading represents the average RNA levels in the WT strain. (C and D) Violin-plot of mean poly(A) tail length distribution of MTREC-associated mRNAs (C) and PROMPTs (D) for the WT and indicated mutant strains. Dots represent the mean poly(A) tail length of individual mRNAs/PROMPTs. (E-H) Box-plots (E,F) and Violin-plots (G, H) of mean poly(A) tail length distribution of MTREC-associated meiotic mRNAs (E, G) and AS RNAs (F, H) for the WT and indicated mutant strains. Dots represent the mean poly(A) tail length of individual meiotic mRNAs/AS RNAs, boxes represent the 25-75 percentile range and the lines represent the median values. Upper and lower whiskers represent 75th percentile plus 1.5 times the inter-quartile distance (IQR) and he 25th percentile minus 1.5IQR, respectively. 5,135 annotated mRNAs, 26 meiotic genes, 619 PROMPTs (FC>2) and 85 AS RNAs (FC>2) are shown. Source data are provided in Source Data file.

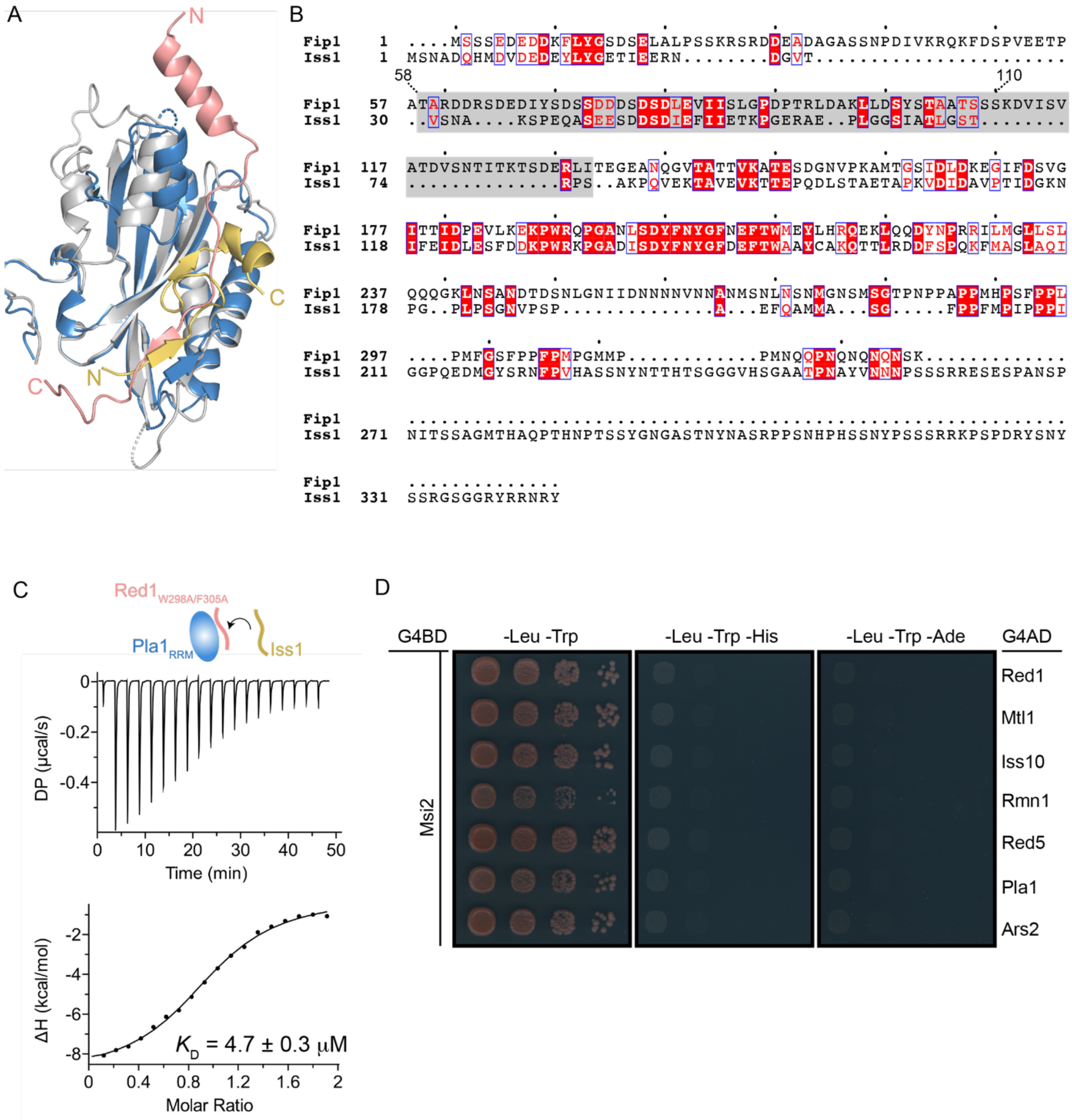

Supplementary Figure 9. Interaction between MTREC and CPF

(A) Structure superposition of *S. cerevisiae* Pap1 (grey) - Fip1 (dark yellow) complex (PDB ID: 3C66) and Pla1 (blue) - Red1 (salmon) complex. Only the RRM domains of Pap1 and Pla1 are shown. (B) Sequence alignment between Fip1 and Iss1. Residues which are identical are highlighted in red while those that are highly similar are colored in red and boxed in blue. Fip1 residues 58-110 shown to be important for binding to Pap1 in *S. cerevisiae*[6] are marked and the corresponding Iss1 residues

30-76 are marked in grey. (C) ITC titration of $Red1_{288-345}$ into a pre-formed complex of $Pla1_{RRM}$-Iss1. The calculated dissociation constants (K_D) from an average of two independent measurements are shown. (D) Y2H experiments do not show interaction between Msi2 and MTREC components apart from Mmi1 and Pab2- related to Figure 7F.

Supplementary Table 1. Summary of dissociation constants measured by ITC

Cell	Syringe	KD (μM)	N value	No. of experiments
$Pla1_{RRM}$	$Red1_{288-345}$	1.56 ± 0.24<br>1.15 ± 0.3<br>1.37 ± 0.22	1.05<br>0.952<br>1.19	3
$Pla1_{RRM}$	$Red1_{288-322}$	7.3 ± 0.1<br>8.4 ± 0.1.4	1.3<br>1.31	2
$Pla1_{RRM}$	$Red1_{288-345}$ W298A/F305A	N.D.	-	2
$Pla1_{RRM}$ K368E	$Red1_{288-345}$	15.3 ± 3.08<br>20.3 ± 19.7	0.8<br>1 (fixed)	2
$Pla1_{RRM}$	$Red1_{288-345}$ V313R	15.8 ± 2.3<br>19.9 ± 2.7	1.13<br>0.96	2
$Pla1_{RRM}$	$Red1_{288-345}$ RSRR	N.D.	-	2
$Pla1_{RRM}$ EEQE	$Red1_{288-345}$	3.28 ± 1.08<br>2.27 ± 0.32	1.03<br>0.97	2
$Pla1_{RRM}$ EEE	$Red1_{288-345}$	9.43 ± 1.9<br>9.98 ± 2.23	0.86<br>0.94	2
$Pla1_{RRM}$	$Red1_{288-345\ S308A}$	1.01 ± 0.28<br>1.45 ± 0.55	1.04<br>1.05	2
$Pla1_{RRM}$	$Red1_{288-345\ N311A}$	0.77 ± 0.21<br>1.53 ± 0.44	0.99<br>1.05	2
$Pla1_{RRM}$	Iss1	2.11 ± 0.12<br>2.94 ± 0.3	1<br>1	2
$Pla1_{RRM}$ + Iss1	$Red1_{288-345}$	9.52 ± 2.83<br>12.4 ± 1.67	1.11<br>1.82	2
$Pla1_{RRM}$ + $Red1_{288-345}$	Iss1	N.D.	-	2
$Pla1_{RRM}$ + $Red1_{288-345}$ W298A/F305A	Iss1	4.58 ± 0.6<br>4.73 ± 0.5	0.97<br>1	2

Supplementary Table 2. SAXS data collection, processing and modeling statistics

	**Pla1-Red1 complex**
**Sample details**	
Source organism	*S. pombe*
Expression organism	*E. coli*
Description: sequence (Uniprot ID)+ uncleaved tags	
Red1	GAMG + Q9UTR8 (288-245) + GSHHHHHH
Pla1	MKHHHHHHP + Q10295 (1-566)
Extinction coefficient ε (M^{-1} cm^{-1})	84340
Molecular mass (kDa)	72.9
**Data collection parameters**	
Instrument	PETRA III, P12 DESY
Beam geometry (mm^2)	0.2 × 0.12
Wavelength (Å)	0.124
q range (nm^{-1})	0.00226 - 7.4054
Exposure time (s)	7.8s (40×0.195s)
Temperature (°C)	20
Concentration range measured (mg ml^{-1})	0.05 - 1.7
Concentration used (mg ml^{-1})	0.85
**Structural parameters**	
R_g (nm) (from $P(r)$)	3.28
R_g (nm) (from Guinier)	3.3±0.02
D_{max} (nm)	10.7
Porod volume estimate (nm^3) (from P(r))	137.79
**Molecular weight determination (kDa)**	
From volume of correlation (V_c)	90.7
From Bayesian assessment [credibility interval], probability	91.175 [86.95, 95.8], 94.6%
Calculated *MW* from sequence	73
**Software employed**	
Primary data reduction	SASFLOW
Data processing	PRIMUS
Flexibility modeling	CORAL

Computation of model intensities	CRYSOL
3D graphics representations	PYMOL
**Data deposition ID**	
Pla1-Red1 complex	SASDKE6

Supplementary Table 3: BLI kinetics experiments

Biotinylated ligand	Analyte	K_D (nM)	k_{on} (1/Ms)	k_{off} (1/s)	χ^2	R^2
$Red1_{288-345}$	$Pla1_{FL}$	59.2±0.57	$2.75*10^5 \pm 2.6*10^3$	$1.63*10^{-2} \pm 2.9*10^{-5}$	2.43	0.9949
Iss1	$Pla1_{FL}$	115.2±1.6	$2.1*10^5 \pm 2.8*10^3$	$2.42*10^{-2} \pm 5.8*10^{-5}$	1.84	0.9946

Supplementary Table 4: *E. coli* expression, yeast two hybrid plasmids and yeast strains used in this study

***E. coli* expression plasmids**

Plasmid No.	Name	Selection	Reference
ND766	pET_His-spPla1-1-566	Kan	this study
ND788	pET_His-spPla1-1-542	Kan	this study
ND1119	pET_His-1a-spPla1-352-566	Kan	this study
ND940	pET_Gb1_CHis-spRed1-240-345	Amp	this study
ND953	pET_Gb1_CHis-spRed1-288-345	Amp	this study
ND954	pET_Gb1_CHis-spRed1-288-322	Amp	this study
ND82	pET_MBP_1a-EYFP	Kan	Gunter Stier
ND769	pET21d_spPla1-352-566	Amp	this study
ND775	pET_MBP_1a- spRed1-240-345	Kan	this study
ND809	pET_MBP_1a- spRed1-259-288	Kan	this study
ND810	pET_MBP_1a- spRed1-288-322	Kan	this study
ND811	pET_MBP_1a- spRed1-288-345	Kan	this study
Rp01	pET_Gb1_CHis-spRed1-RSRR	Amp	this study
Rp02	pET_Gb1_CHis-spRed1-S308A	Amp	this study

Rp04	pET_Gb1_CHis-spRed1-WFAA	Amp	this study
Rp05	pET_Gb1_CHis-spRed1-N311A	Amp	this study
Rp10	pET_His-1a-spPla1-EEQE	Kan	this study
Rp11	pET_His-1a-spPla1-EEE	Kan	this study
Rp12	pET_His-spPla1-D153A	Kan	this study
Rp17	pET_Gb1_CHis-spRed1-V313R	Amp	this study
Rp18	pET_His-1a-spPla1-K368E	Kan	this study

Yeast two hybrid plasmids

Plasmid No.	Name	Selection	Reference
ND333	pGBKT7	Kan	Dobrev et al.[7]
ND334	pGADT7	Amp	Dobrev et al.[7]
ND368	pGADT7-spRed1_1-712	Amp	Dobrev et al.[7]
ND370	pGADT7-spMtl1_1-1030	Amp	Dobrev et al.[7]
ND435	pGADT7 -spRed5_1-376	Amp	Dobrev et al.[7]
ND436	pGADT7 -spIss10_1 -551	Amp	Dobrev et al.[7]
ND437	pGADT7 -spMmi1_1-488	Amp	Dobrev et al.[7]
ND438	pGADT7 -spPab2_1 -166	Amp	Dobrev et al.[7]
ND439	pGADT7 -spRmn1_1 -590	Amp	Dobrev et al.[7]
Rp19	pGADT7 -spPla1_1 -566	Amp	this study
Rp20	pGADT7 -spArs2_1-609	Amp	this study
Rp21	pGBKT7 -spMsi2_1 -474	Kan	this study
Rp15	pGADT7-spRed1_1-712-WFAA	Amp	this study
Rp16	pGADT7-spRed1_del288-345	Amp	this study

Yeast strains

Strain No.	Name	Relevant Genotype	Reference
P419	WT	*MatMsmt0, leu1-32, ura4-D18, ade6-216*	

P1	WT	*MatMsmt0, leu1-32, ura4 DS/E, ade6-210, his2, Otr1R:: Ura4 (SphI)*	
P344	WT	*h+, HIS, leu1-32, ade6-210, ura4-D18*	V2-33-H12, Bioneer Inc
F3230	Red1 (WT)	*MatMsmt0, leu1-32, ura4-D18 ade6-M210, Red1:: natNT2 (+3'UTR)*	this study
F3232	$red1_{W298A/F305A}$	*MatMsmt0, leu1-32, ura4-D18 ade6-M210/M216,* $Red1_{W298A/F305A}$ *:: natNT2 (+3'UTR)*	this study
F3436	$red1_{\Delta 288-345}$	*MatMsmt0, leu1-32, ura4-D18, ade6-M210,* $Red1_{\Delta 288-345}$ *:: natNT2 (+3'UTR)*	this study
F3237	*red1Δ*	*MatMsmt0, leu1-32, ura4-D18, ade6-M210, red1Δ:: kanMX6*	this study
F3269	Red1-FTP	*MatMsmt0, leu1-32, ura4-D18, ade6-M216, Red1-3xFTP:: hyg*	this study
F3271	$red1_{W298A/F305A}$*-FTP*	*MatMsmt0, leu1-32, ura4-D18, ade6-M210,* $Red1_{W298A/F305A}$*-3xFTP::hyg*	this study
F3273	$red1_{\Delta 288-345}$*-FTP*	*MatMsmt0, leu1-32, ura4-D18, ade6-M216,* $Red1_{\Delta 288-345}$*-3xFTP::hyg*	this study
F3043	Red1-FTP	*h90, leu1-32, ura4-D18, ade6-M210, Red1- 3xFTP::hyg.*	this study
F3197	$red1_{W298A/F305A}$*-FTP*	*h90, leu1-32, ura4-D18, ade6-M210* $Red1_{W298A/F305A}$*-3xFTP::hyg.*	this study

F3199	*$red1_{\Delta 288\text{-}345}$-FTP*	*h90, leu1-32, ura4-D18 ade6-M210, $Red1_{\Delta 288\text{-}345}$-3xFTP::hyg.*	this study
F3203	Pla1-HA	*h+, HIS, leu1-32, ade6-210, ura4-D18 Pla1-3xHA::kanMX6*	this study
F3205	Red1-FTP; Pla1-HA	*h90, leu1-32, ura4-D18, ade6-M210 Red1-3xFTP::hyg; Pla1-3xHA::kanMX6*	this study
F3207	*$red1_{W298A/F305A}$-FTP*; Pla1-HA	*h90, leu1-32, ura4-D18, ade6-M210 $Red1_{W298A/F305A}$-3xFTP::hyg; Pla1- 3xHA::kanMX6*	this study
F3209	*$red1_{\Delta 288\text{-}345}$-FTP*; Pla1-HA	*h90, leu1-32, ura4-D18, ade6-M210 $Red1_{\Delta 288\text{-}345}$-3xFTP::hyg; Pla1-3xHA::kanMX6*	this study

Supplementary Table 5: Primers used in this study

**Primer No.**	***Name***	**Sequence (5'->3')**
ND342	*spPla1-NcoI-FW*	TATACCATGGGTACTACCAAGCAATGGGGTATTACAC
ND343	*spPla1-BamHI-RE*	TATAGGATCCTTATGCCGTTGAAACTTTTTGTCGTTTTAATTG
ND432	*spPla1-352-NcoI-FW*	TATACCATGGACTTTTTTCATCGTTATAAGCATTATC
ND433	*spPla1-352-XhoI-RE*	TATACTCGAGTTAGTCGTGTTTTTGAAACAAAGCTGAC
N748	*spPla1-542-STOP-BamHI-RE*	TATAGGATCCTTAAGCTTTCGGCCGTTCTTCTCC

N620	*spRed1_288-FW-GA-BamHI*	GAAGAACGGCCGAAAGCTGGTGGTTCTGGCGGTTCCATATCTTTACCACTTTTGAAGCAGG
N619	*SpRed1_345-RE-GA-BamHI*	GTCAGTGGTGGTGGTGGTGGTGGGATCCATTTTCACTATTTGAGGGGG
N621	*SpRed1_322-RE-GA-BamHI*	GTCAGTGGTGGTGGTGGTGGTGGGATCCATCATCATCGGAATCAAATTCAATAAC
KS1	*spRed1_F305A_FP*	CGATTGGCTATCTTCTTCGAAGCCTGCTGGCTCCTCGAC
KS2	*spRed1_F305A_RP*	AGTCGAGGAGCCAGCAGGCTTCGAAGAAGATAGCCAATCG
KS3	*spRed1_W298A_FP*	CCACTTTTGAAGCAGGACGATGCGCTATCTTCTTCGAAGC
KS4	*spRed1_W298A_RP*	CTTCGAAGAAGATAGCGCATCGTCCTGCTTCAAAAGTGG
KS5	*spRed1_N311A_FP*	GCCTTTTGGCTCCTCGACTCCAGCTGTAGTTATTGAATTTGATTC
KS6	*spRed1_N311A_RP*	GAATCAAATTCAATAACTACAGCTGGAGTCGAGGAGCCAAAAGGC
KS7	*spRed1_D317RRR_FP*	CTTTTGGCTCCTCGACTCCAAATGTAGTTATTGAATTTCGTTCCCGTCGTGATGGAGATGACTTTTCGA
KS8	*spRed1_D317RRR_RP*	TCGAAAAGTCATCTCCATCACGACGGGAACGAAATTCAATAACTACATTTGGAGTCGAGGAGCCAAAAG
KS9	*spRed1_S308A_FP*	CGAAGCCTTTTGGCTCCGCGACTCCAAATGTAGTT
KS10	*spRed1_S308A_RP*	AACTACATTTGGAGTCGCGGAGCCAAAAGGCTTCG
KS11	*spPla1-K368E-RP*	GAGCTTCAGCTGTCTCAGCAGCTGCTGTGATCG
KS12	*spPla1-K368E-FP*	CGATCACAGCAGCTGCTGAGACAGCTGAAGCTC
KS13	*spPla1_EEE_FP*	AAGAACGGCCGAAAGCTACTGAGGAGGAGTCCACTGCTGACACTGCAC
KS14	*spPla1_EEE_RP*	GTGCAGTGTCAGCAGTGGACTCCTCCTCAGTAGCTTTCGGCCGTTCTT

KS15	*spPla1_EEQE_BamHI_RP*	CATGTCGGATCC TTA TGCCGTTGAAACTTCTTGTTCTTCTAATTGCTCTGTACT ATG
KS16	*spPla1_352_NcoI_FP*	GAGACTACC ATGG ACTTTTTTCATCGTTATAAGCATTATCTTAC
KS17	*spPla1_D153A_FP*	AGTTTAAATTTTTGGGAATAAGTATAGCTTTAATTTTTGC TCGTCTTTCAGTTCC
KS18	*spPla1_D153A_RP*	GGAACTGAAAGACGAGCAAAAATTAAAGCTATACTTATT CCCAAAAATTTAAACT
KS19	*spRed1-V313R-RP*	CATCGGAATCAAATTCAATACGTACATTTGGAGTCGAG GAGCC
KS20	*spRed1-V313R-FP*	GGCTCCTCGACTCCAAATGTACGTATTGAATTTGATTC CGATGA
KS21	*spIss1-30-NcoI-FP*	GAGACTACC ATG G TGTCAAATGCCA AGTCACCAGA
KS22	*spIss1-76-BamHI-RP*	TATAGGATCCTTAAGAAGGTCGGGTTGAACCTAATGTT
KS23	*spRed1-del288-345-RP*	GTAATCTGAACGAGACATAGTAAGAAGATTTTTTCTTTC AGGAAGGACG
KS24	*spRed1-del288-345-FP*	CGTCCTTCCTGAAAGAAAAAATCTTCTTACTATGTCTCG TTCAGATTAC
KS25	*spMsi2_NcoI_FW-GBKT7--GA*	AGGAGGACCTGCATATGGCCATGGGCGGCTCTGATTT TGAAGATG
KS26	*spMsi2_BamHI_RE-GBKT7-GA*	TGCAGGTCGACGGATCCTTATCGACGATATGGATGGAA GCTGTGCC
KS27	*spPla1_NcoI_FW-ADT7-GA*	GGCGAGCGCCGCCATGACTACCAAGCAATGGGGTATT ACAC
KS28	*spPla1_BamHI_RE-ADT7-GA*	GCAGCTCGAGCTCGATGGATCCTTATGCCGTTGAAACT TTTTGTCGTTTTAATTG

KS29	*spArs2_NcoI_FW-ADT7-GA*	GGCGAGCGCCGCCATGGCTAGTGAGGTCCATCAAGAAAG
KS30	*spArs2_BamHI_RE-ADT7-GA*	GCAGCTCGAGCTCGATGGATCCTTAGTAATCTAATTCTGGAACCTCCTGGTTAG
AN77	*spRed1_For_ F305A*	GCGCTATCTTCTTCGAAGCCTGCTGGCTCCTCGACTCCAAATG
AN78	*spRed1_Rev_W298A*	GCAGGCTTCGAAGAAGATAGCGCATCGTCCTGCTTCAAAAGTGG
AN80	*spRed1_ For_Nhe1 site*	CGGACTGGATTCCTCTTCTGCTAGCTCCCAAGGAAGTCTGAC
AN70	*spRed1-Pla1 DEL_Rev_first frag*	CTGAACGAGACATAGTAAGACCAAGATTTTTTCTTTCAGGAAGG
AN81	*spRed1_Rev with Kpn1 site*	GATATAATCGACAAGCGGTACCTTATCCTCAAAATCTGTTTCCGTC
AN82	*spRed1_For DEL sec_ frag.*	CGTCCTTCCTGAAAGAAAAAATCTTGGTCTTACTATGTCTCGTTCAG
AN62	*spRed1_Fw_above 400bp*	CGCTTGTCGATTATATCTCTC
AN63	*spRed1_Rev_natNT2OH*	CGTCGACCTGCAGCGTACGTTATCAATAACCATTGAAATG
AN64	*spRed1_Fw_ Clonat Overhang*	CGAGCTCGAATTCATCGATAAATGCGCGTATTGTAATAAG
AN65	*spRed1_ Rev. 500bp after.*	GTTTGGAAGATGGTTTACGCCTAC
AN86	*Pla1-3xHA tag first frag._For.*	GCTCAACAAGTAGCTAGCGG
AN87	*Pla1-3xHA tag first frag._Rev.*	GTTAATTAACCCGGGGATCCGTGCCGTTGAAACTTTTTGTC
AN88	*Pla1-3xHA tag sec. frag._For.*	CGAGCTCGAATTCATCGATGTAAATACCCACACATAAGACTC
AN89	*Pla1-3xHA tag sec. frag._Rev.*	GAACACTCCTAAAGATCCCAAC
AN139	*ChIP_check_ura4DS/E#1*	GAGGGGATGAAAAATCCCAT

AN140	*ChIP_check_ura4DS/E#2*	TTCGACAACAGGATTACGACC
AN162	*GAPDH_For.*	AACATCATCCCCTCCTCCAC
AN163	*GAPDH_Rev.*	GCCTTGATGTCCTCGTAGTTG

Supplementary References:

1. Buchan, D.W.A. & Jones, D.T. The PSIPRED Protein Analysis Workbench: 20 years on. *Nucleic Acids Res* **47**, W402-W407 (2019).
2. Shen, Y., Delaglio, F., Cornilescu, G. & Bax, A. TALOS+: a hybrid method for predicting protein backbone torsion angles from NMR chemical shifts. *J Biomol NMR* **44**, 213-23 (2009).
3. Petoukhov, M.V. et al. New developments in the ATSAS program package for small-angle scattering data analysis. *J Appl Crystallogr* **45**, 342-350 (2012).
4. Franke, D. et al. ATSAS 2.8: a comprehensive data analysis suite for small-angle scattering from macromolecular solutions. *J Appl Crystallogr* **50**, 1212-1225 (2017).
5. Mirdita, M. et al. ColabFold: making protein folding accessible to all. *Nat Methods* **19**, 679-682 (2022).
6. Kumar, A. et al. Dynamics in Fip1 regulate eukaryotic mRNA 3' end processing. *Genes Dev* (2021).
7. Dobrev, N. et al. The zinc-finger protein Red1 orchestrates MTREC submodules and binds the Mtl1 helicase arch domain. *Nat Commun* **12**, 3456 (2021).
